# Morphometric analyses and gene expression related to germ cells, gonadal ridge epithelial-like cells and granulosa cells during development of the bovine fetal ovary

**DOI:** 10.1371/journal.pone.0214130

**Published:** 2019-03-22

**Authors:** Katja Hummitzsch, Nicholas Hatzirodos, Helen F. Irving-Rodgers, Monica D. Hartanti, Viv E. A. Perry, Richard A. Anderson, Raymond J. Rodgers

**Affiliations:** 1 Discipline of Obstetrics and Gynaecology, School of Medicine, Robinson Research Institute, University of Adelaide, Adelaide, South Australia, Australia; 2 School of Medical Science, Griffith University, Gold Coast Campus, Gold Coast, Queensland, Australia; 3 School of Veterinary Medicine and Science, University of Nottingham, Sutton Bonington, Leicestershire, United Kingdom; 4 Medical Research Council Centre for Reproductive Health, University of Edinburgh, Queen’s Medical Research Institute, Edinburgh, United Kingdom; Peking University Third Hospital, CHINA

## Abstract

Cells on the surface of the mesonephros give rise to replicating Gonadal Ridge Epithelial-Like (GREL) cells, the first somatic cells of the gonadal ridge. Later germ cells associate with the GREL cells in the ovigerous cords, and the GREL cells subsequently give rise to the granulosa cells in follicles. To examine these events further, 27 bovine fetal ovaries of different gestational ages were collected and prepared for immunohistochemical localisation of collagen type I and Ki67 to identify regions of the ovary and cell proliferation, respectively. The non-stromal cortical areas (collagen-negative) containing GREL cells and germ cells and later in development, the follicles with oocytes and granulosa cells, were analysed morphometrically. Another set of ovaries (n = 17) were collected and the expression of genes associated with germ cell lineages and GREL/granulosa cells were quantitated by RT-PCR. The total volume of non-stromal areas in the cortex increased significantly and progressively with ovarian development, plateauing at the time the surface epithelium developed. However, the proportion of non-stromal areas in the cortex declined significantly and progressively throughout gestation, largely due to a cessation in growth of the non-stroma cells and the continued growth of stroma. The proliferation index in the non-stromal area was very high initially and then declined substantially at the time follicles formed. Thereafter, it remained low. The numerical density of the non-stromal cells was relatively constant throughout ovarian development. The expression levels of a number of genes across gestation either increased (*AMH*, *FSHR*, *ESR1*, *INHBA)*, declined (*CYP19A1*, *ESR2*, *ALDH1A1*, *DSG2*, *OCT4*, *LGR5*) or showed no particular pattern (*CCND2*, *CTNNB1*, *DAZL*, *FOXL2*, *GATA4*, *IGFBP3*, *KRT19*, *NR5A1*, *RARRES1*, *VASA*, *WNT2B*). Many of the genes whose expression changed across gestation, were positively or negatively correlated with each other. The relationships between these genes may reflect their roles in the important events such as the transition of ovigerous cords to follicles, oogonia to oocytes or GREL cells to granulosa cells.

## Introduction

The ovary development starts with the formation of the genital ridge formed by increased proliferation of the surface epithelium on the ventromedial side of the mesonephros. These proliferating cells are now termed the gonadal ridge epithelial-like (GREL) cells [1] and express cytokeratin 19 (*KRT19*) and desmosomal proteins such as desmoglein-2 (*DSG2*) and plakophilin-2. Later in development the GREL cells differentiate into pre-granulosa or surface epithelial cells. The formation of the genital ridge requires the expression of empty spiracles homeobox protein 2 (*EMX2*), LIM homeobox protein 9 (*LHX9*), Wilms Tumor 1 (*WT1*), steroidogenic factor (*SF1*/*NR5A1*) [2, 3] and GATA binding protein 4 (*GATA4*) [4]. The absence of sex determining region Y (*SRY*) results in the bipotential gonad committing to the ovary-determining pathway. This is accompanied by the expression of Wingless-related MMTV integration site family member 4 (*Wnt4*) [5], β-catenin (*CTNNB1*) [6], R-spondin 1 (*Rspo1*) [7] and Forkhead box L2 (*FOXL2*) [8, 9] [10].

The primordial germ cells arise from the yolk sac and migrate under the influence of kit ligand and its receptor towards the gonad. After colonising the developing gonad composed of GREL cells, the primordial germ cells start proliferating and express pluripotency markers such as octamer binding protein 4 (*OCT4*) [11]. Later in the developing ovary the mitotically active germ cells, termed oogonia, become oocytes after entering meiosis. The entry into meiosis is accompanied by expression of deleted in azoospermia-like (*DAZL*) and induced by retinoic acid, which is synthesised by aldehyde dehydrogenases (*ALDH1-3*) [12]. Subsequently germ cells switch from expressing *DAZL* to *VASA* [11]. Oocytes arrest in the dictyate phase of meiosis I until shortly before ovulation when meiosis is resumed.

The ovarian stroma arises from the mesonephric connective tissue after breakdown of the basal lamina underlying the surface epithelium [1]. This stroma, including its vasculature, penetrates the mass of GREL cells and PGCs/oogonia, branching as it does and so corralling the GREL and germ cells into forming the ovigerous cords [1]. Subsequently the continued expansion of the stroma [13] likely separates the ovigerous cords into smaller cords until the first primordial follicles are formed, consisting of one layer of flattened pre-granulosa cells and a meiotically-arrested oocyte [1, 14, 15]. In the mouse, two different populations of primordial follicles have been identified [16]. Medullary follicles are activated shortly after birth, while cortically located follicles activate gradually throughout life. In addition, medullary pre-granulosa cells express *FOXL2* while cortical pre-granulosa cells express Leucine Rich Repeat Containing G Protein-Coupled Receptor 5 (*LGR5*) [17].

Lateral spread of the stroma below the surface of the ovary partitions some GRELs to the surface, which become the surface epithelial cells. Fibroblasts, the major cell type of the stroma, express Nuclear Receptor Subfamily 2 Group F Member 2 (*NR2F2/COUP-TFII*) and produce fibrillar extracellular matrix components, such as collagen type I (*COL1A1*), collagen type III (*COL3A1*) and fibrillins (*FBN1-3*) [1, 18]. Members of the TGFβ-superfamily have important roles during oogenesis and folliculogenesis [19–22]. Activin or inhibin (*INHBA*), anti-Mullerian hormone (*AMH*) and FSH receptor (*FSHR*) are involved in granulosa cell proliferation and differentiation [19].

Many of the recent discoveries of the developing ovary come from studies of mice using lineage tracing techniques and manipulation of gene expression during development. Whilst these have helped to make substantial gains on our knowledge, these studies generally have not fully assessed the behaviours of the different somatic cell types, such as GREL cells and fibroblasts, and the processes that these can undertake in unison at different stages of ovarian development. To address this, the current study first examined replication of non-stromal cells and the changes in their volume during gestation and compares this with changes in the stromal compartments [13]. The expression patterns of genes related to germ and stem cells, and GREL and granulosa cells in fetal ovaries across gestation were also examined and discussed in relation to changes occurring in the ovary at those times. Genes related to stromal cells and the extracellular matrix have been discussed in a companion publication [23].

## Materials and methods

### Tissues

Fetuses of pregnant *Bos taurus* cows were collected at T&R Pastoral abattoir at Murray Bridge, SA, Australia and then transported on ice to the laboratory. Crown-rump length was measured to estimate gestational age [24]. Some ovaries were fixed in 4% paraformaldehyde (Merck Pty Ltd, Kilsyth, VIC, Australia) in 0.1 M phosphate buffer (pH 7.4) for immunohistochemistry and morphometric analyses (n = 27) and others from different animals were frozen at -80°C for subsequent RNA analyses (n = 17).

### Gender determination

To confirm the gender of young fetuses (smaller than 8 cm), genomic DNA was extracted from the tail samples using the Wizard SV Genomic DNA Purification System (Promega Australia, Alexandria, NSW, Australia) according to the manufacturer’s instructions. Genomic DNA was amplified with a primer pair (sense primer: 5’-TCACTCCTGCAAAAGGAGCA-3’, antisense primer: 5’-TTATTGTGGCCCAGGCTTG-3’), specific for a region in the SRY-determining sequence, and primers specific for the 18S ribosomal RNA gene sequence [25] in separate reactions. SRY product sequences were verified by automated sequencing (3730 DNA analyser; Applied Biosystems, Mulgrave, VIC, Australia).

### Histology and immunohistochemistry

Fixed ovaries, as used previously [13] were embedded in paraffin using a Leica EG 1140H (Leica Microsystems, Nussloch, Germany). Six-μm sections were cut using a CM1850 V2.2 Leica microtome (Leica Microsystems), mounted on Superfrost glass slides (HD Scientific Supplies, Wetherill Park, NSW, Australia) and stored at RT until used for haematoxylin-eosin (H&E) staining and immunohistochemistry. H&E-stained sections were used for sample grouping based on histological morphology: stage 1—ovigerous cord formation (n = 7, 79 ± 6 days of gestation), stage 2—ovigerous cord breakdown (n = 4, 127 ± 6 days), stage 3—follicle formation (n = 3, 173 ± 12 days), stage 4—ovarian surface epithelium formation (n = 8, 234 ± 9 days) and stage 5—tunica albuginea formation (n = 5, 264 ± 6 days)] [13].

**Fig 1:**
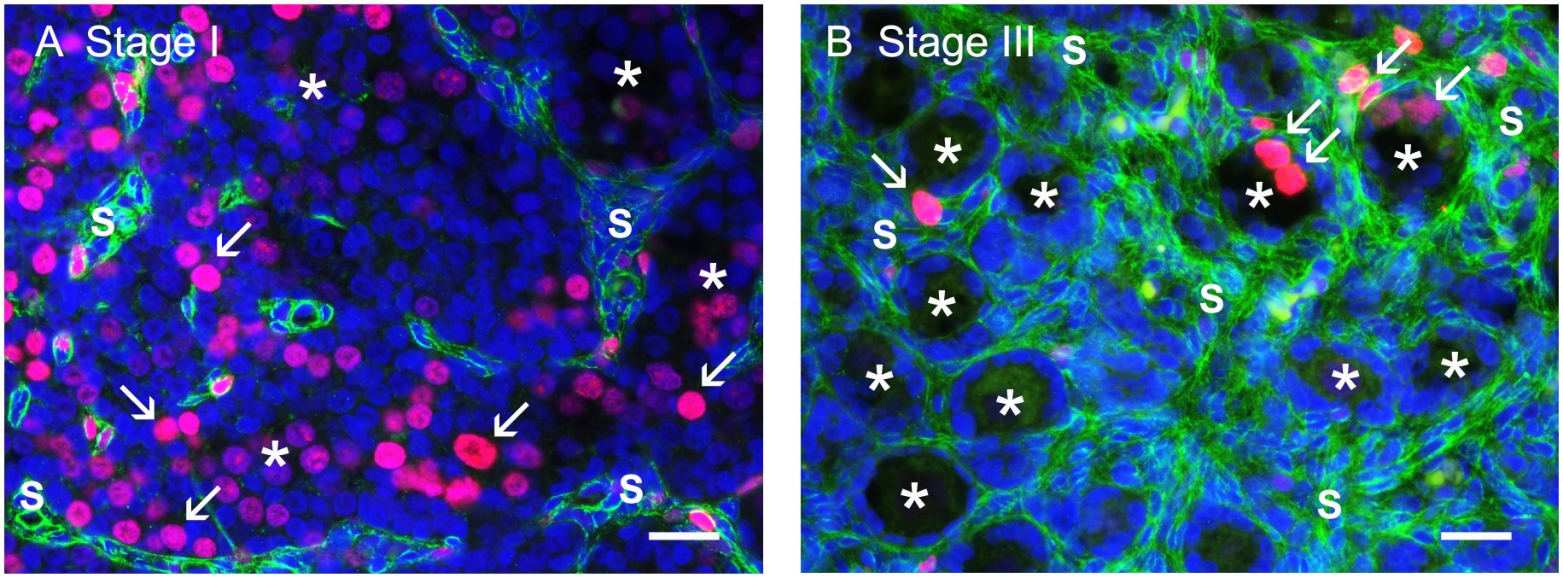
Representative photographs from stage I (the formation of ovigerous cords) (A) and stage III (the formation of follicles) (B). Collagen type I (green) is colocalised with the proliferation marker Ki67 (red, marked with arrow). Nuclei are counterstained with DAPI. Scale bar: 25 μm. * ovigerous cords/ primordial follicles, S stroma.

An indirect immunofluorescence method was used for colocalisation of Ki-67 and collagen type I as previously described in detail previously [13] and illustrated in Fig 1. Primary antibodies were mouse anti-human Ki67 (1:800; M7240/MIB-1; DAKO Australia Pty Ltd, Botany, NSW, Australia) to identify proliferating cells in combination with rabbit anti-human collagen type I (1:400; 20μg/ml; ab34710; Abcam, Sapphire Bioscience Pty Ltd., Waterloo, NSW, Australia) to identify the stroma. Secondary antibodies were donkey anti-mouse IgG conjugated to Cy3 (1:100; 715-166-151) and biotin-SP-conjugated AffiniPure donkey anti-rabbit IgG (1:100; 711-066-152) followed by fluorescein DTAF-conjugated streptavidin (1:100; 016-010-084). All secondary antibodies and conjugated streptavidin were from Jackson ImmunoResearch Laboratories Inc. (West Grove, PA, USA). Cell nuclei were counterstained with 4’,6’-diamidino-2-phenylindole dihydrochloride (DAPI) solution (Molecular Probes, Eugene, OR, USA). Non-immune mouse and rabbit sera (Sigma-Aldrich, New South Wales, Australia) were used as negative controls. All sections were photographed with an Olympus BX50 microscope (Olympus, Tokyo, Japan) with an epifluorescence attachment and a Spot RT digital camera (Diagnostic Instruments, Sterling Heights, MI, USA) at a magnification of 40x.

### Image analyses

Fluorescence images were analysed with ImageJ software. For this purpose, the cortical region of the largest section from each ovary was examined morphometrically. Ten fields of view were analysed for ovaries from fetuses with CRL < 50 cm (182 days of gestation) and 20 fields of view (0.06 mm^2^) for a CRL > 50 cm. The image analysis procedure has been described in detail by Hartanti et al. [13]. To identify the amount of non-stromal compartment in the cortex containing the ovigerous cords and follicles, the stromal area identified by collagen type I staining was subtracted from the total cortical area. The proportion of non-stromal in the cortex (volume density) was calculated as the ratio between the non-stromal area and the total cortical area. The ovarian volume was estimated using the ovarian weight and assuming a density of 1g/cm^3^. The volume of the cortex was then derived from the volume of the ovary and the volume density of the cortex. Similarly, the non-stromal volume was calculated from the volume of cortex and the volume density of non-stroma in the cortex. The number of proliferating cells (KI-67 positive) in ovigerous cords or follicles and the total number of non-stromal cells (DAPI positive) in a field of view were counted. Results of proliferating cells were presented as a proliferation index and as a numerical density in the non-stromal area.

### RNA extraction, cDNA synthesis and quantitative real time PCR

All fetal ovary samples for RNA sample extraction were homogenised with 1 ml of Trizol (Cat # 15596–026, Thermo Fisher Scientific, Waltham, MA, USA) each for two 10 s cycles at 3,500 rpm in a PowerLyzer 24 Bench Top Bead-Based Homogeniser (MoBio, Carlsbad, Ca, USA). The samples were then processed according to the standard Trizol protocol and resuspended in 30 μl of nuclease free H_2_O. Ten μg or less of each sample was treated with 2 U of DNAse 1 for 20 min at 37° C and the enzyme removed using DNAse inactivation reagent (Thermo Fisher Scientific). Two hundred ng of DNAse-treated RNA was used for reverse transcription reactions with or without Superscript RT III (Thermo Fisher Scientific) to generate cDNA or negative control to detect genomic contaminant, respectively.

PCR primers pairs were designed where possible to span intron-exon junctions or from different exons for quantitative real time PCR (qRT-PCR) using the free web-based software programs, Primer3 plus (Rozen and Skaletsky) and Net primer (PREMIER Biosoft, Palo Alto, CA, USA), based on the Reference RNA sequences available in NCBI (Table 1). The cDNA was diluted from 1 in 4 to 1in 1000 and pooled from 10 samples to generate 5 standards which were used to establish a standard curve for testing primer combinations for quantitative real time PCR. Only those assays which gave a single sharp peak by melt curve analysis and achieved an amplification efficiency of 0.9–1.1 and an R^2^ value ≥ 0.98 were used for quantitation of gene expression.

**Table 1:** List of genes and primers used for qRT-PCR.

Gene name	Gene Symbol	GenBank Accession No.	Primers (5’-3’)	Size (bp)
Aldehyde dehydrogenase 1 family, member A1	*ALDH1A1*	NM_174239.2	F: GCGGAAACACAGTGGTTGTC	150
			R: GAGAAGAAATGGCTGCCCCT	
Anti Mullerian Hormone	*AMH*	NM_173890.1	F: ACACCGGCAAGCTCCTCAT	67
			R: CACCATGTTTGGGACGTGG	
Cyclin D2	*CCND2*	NM_001076372.1	F: GGTGGATCTCCTGGCAAAGA	98
			R: ACGGTACTGCTGCAGGCTATTC	
Catenin (cadherin-associated protein), beta 1, 88kDa	*CTNNB1*	NM_001076141	F: GAATTGACAAAACTGCTGAATGATG	102
			R: GATGGCGTGTCTCGAAGCTT	
Cytochrome P450 family 19 subfamily A, member 1	*CYP19A1*	NM_174305.1	F: ATGCTTTTGGAAGTGCTG	143
			R: TTAGCGCTCGAGGCAC	
Deleted in azoospermia-like	*DAZL*	NM_001081725.1	F: ACGTTTTGCCCAGTGAATGC	98
			R: TACCACCGTCTGTATGCTTCTG	
Desmoglein 2	*DSG2*	NM_001192172.2	F: AAGACCCTCGCTGAAGTTTG	84
			R: TGCTTCTCTTGCGGGTTTTG	
Estrogen receptor 1	*ESR1*	NM_001001443.1	F: GTCCACCTTTTGGAATGTGC	124
			R: ATTTTCCCTGGTTCCTGTCC	
Estrogen receptor 2	*ESR2*	NM_174051.3	F: TCGACTTCGGAAGTGCTATGAG	136
			R: ACCGTTCCTCTTGGTTTTGC	
Forkhead box L2	*FOXL2*	NM_001031750.1	F: AGAATAGCATCCGCCACAAC	127
			R: CCCTTCTCGAACATGTCCTC	
Follicle stimulating hormone receptor (FSHR)	*FSHR*	NM_174061.1	F: GACCCTGATGCCTTCCAGA	74
			R: TGGCAAGTGCTTAATACCTGTGTT	
Glyceraldehyde 3-phosphate dehydrogenase	*GAPDH*	NM_001034034.2	F: ACCACTTTGGCATCGTGGAG	76
			R: GGGCCATCCACAGTCTTCTG	
GATA binding protein 4	*GATA4*	NM_001192877.1	F: CAGGAGGCAAAAATGCTAGG	82
			R: ATCACCCGTCGTCTTTCTTC	
Inhibin, beta A	*INHBA*	NM_174363.2	F: ATCATCACGTTCGCGGAATC	144
			R: ACTTTGCTCCGGGTCCTGTT	
Keratin 19	*KRT19*	NM_001015600.3	F: AAGCTTTGCGCATGAGTGTG	97
			R: TCAATCTGCATCTCCAGGTCAG	
Leucine-rich repeat containing G protein-coupled receptor 5	*LGR5*	NM_001277226	F: TTGGGAGATCTGCTTTTCAACA	64
			R: TGTGAGGCGCCATTCAAA	
Nuclear receptor subfamily 5, group A, member 1	*NR5A1*	NM_174403.2	F: CAGACCTTCATCTCCATCGTG	147
			R: CTTGCCATGCTGAATCTGAC	
POU class 5 homeobox 1	*OCT4*	NM_174580.2	F: AGGCTTTGCAGCTCAGTTTC	79
			R: TTGTTGTCAGCTTCCTCCAC	
Ribosomal protein L19	*RPL19*	NM_001040516.1	F: GATCCGGAAGCTGATCAAAG	113
			R: TACCCATATGCCTGCCTTTC	
DEAD (Asp-Glu-Ala-Asp) box polypeptide 4	*VASA*	NM_001007819.1	F: ATGAAGCTGATCGCATGCTG	91
			R: TGACGCTGTTCCTTTGATGG	
Wingless-type MMTV integration site family, member 2B	*WNT2B*	NM_001099363	F: CGGACTGACCTGGTCTACTTTG	67
			R: AGGGAACCTGCAGCCTTGT	

F is forward and R is the reverse primer sequence.

The reactions were performed in duplicate on a Fluidigm Biomark HD instrument (San Francisco, CA, USA) using the following protocol. The reaction started with a pre-amplification step consisting of a 95°C hold for 10 min, followed by 12 cycles of 95°C for 15 s and 60°C for 4 min each using 50 nM of each primer and 2.5 μl of cDNA in 10 μl. The amplified product was then diluted 1 in 5 and added in 0.05 μl to the final reaction volume of 0.1 μl in a 48 x 48 plate which contained 500 nM of each primer per assay. The final amplification conditions were a 60 s activation step at 95°C, followed by 30 cycles of 96°C denaturation for 5 s and 60°C annealing/extension for 20 s using SsoFast EvaGreen Supermix with Low ROX (Biorad, Hercules, Ca, USA) which contained a fluorescent intercalating agent for measuring amplification. The expression values for each gene were determined as the mean of the ratio of 2^-Δ Ct^ for the target gene to the mean of *RPL32* and *PPIA* because this gene combination was determined to be the most stable across all samples out of *RPL32*, *PPIA*, *ACTB* and *GAPDH* with a value of 0.056 using the Normfinder program.

### Statistical analyses

ANOVA and post-hoc statistical calculations using Tukey’s test were performed using GraphPad Prism version 6.00 (GraphPad Software Inc., La Jolla, CA, USA) following log transformation where appropriate to normalise the raw data distribution. Pearson’s correlations across all target genes and samples were performed on the data in Partek Genomics Suite (St Louis, MI, USA). Hierarchical clustering by gene only was also performed on the data using the Euclidian algorithm for dissimilarity with average linkage in Partek to generate a heatmap of relative gene expression. Prior to clustering, the raw data were first normalised by adjusting the mean expression across all samples for each gene to zero and the standard deviation to one.

## Results and discussion

### Morphometric analyses of non-stromal component of the ovarian cortex

**Fig 2:**
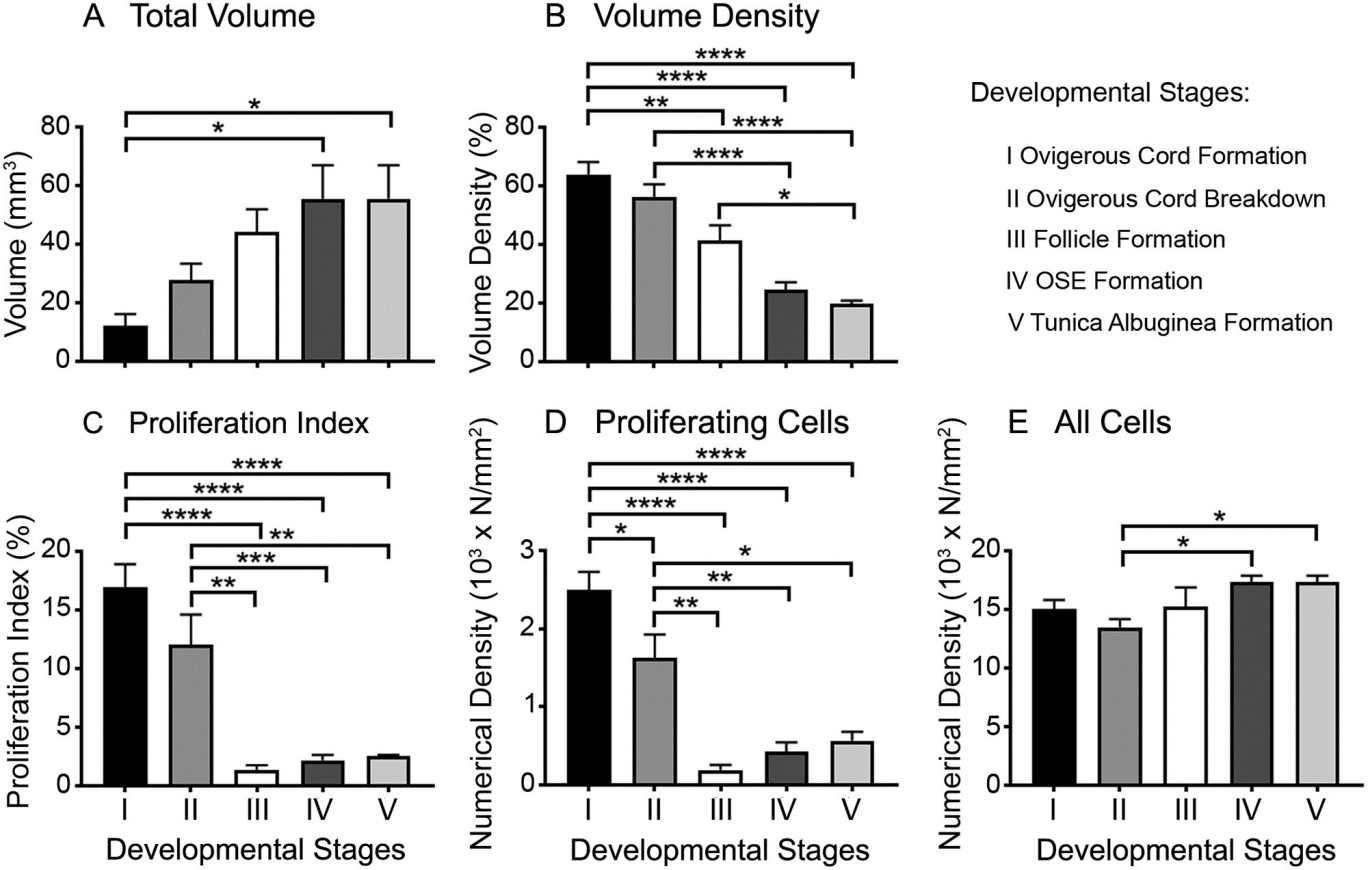
Morphometric analyses of the non-stromal component of the ovarian cortex during bovine fetal ovarian development. Data are presented as means ± SEM. Samples were grouped into 5 stages of ovarian development based on their histological morphology: ovigerous cord formation (n = 7, stage I), ovigerous cord breakdown (n = 4, stage II), follicle formation (n = 3, stage III), ovarian surface epithelium (OSE) formation (n = 8, stage IV) and tunica albuginea formation (n = 5, stage V). One-way ANOVA with post hoc Tuckey’s test were used to analyse the data. * *P* < 0.05, ** *P* < 0.01, *** *P* < 0.001, **** *P* < 0.0001. Volume density is the percentage of cortex that is the non-stromal compartment.

The non-stromal area of the cortex includes ovigerous cords at early stages (stages I and II) and follicles at later stages (stages III to V) (Fig 1). Its total volume increased significantly and progressively with ovarian development but plateaued at stage IV (Fig 2A). However the proportion of non-stroma in the cortex (volume density) declined significantly and progressively throughout gestation (Fig 2B), largely due to a cessation at stage III as indicated by the plateauing in its volume, and the continued growth of stroma after stage III [13].

Expression of Ki67 was used to estimate the percentage of proliferating cells at different developmental stages. The proliferation index of the non-stromal area was highest at stages I and II, declining substantially at stage III and remaining very low thereafter (Fig 2C). Furthermore, we calculated the numerical density of proliferating cells and all cells in the non-stromal compartment. The numerical density of proliferating cells also significantly declined at stage III and then remained low (Fig 2D). The observed high proliferation in the early stages of ovarian development relates mainly to the germ cells (identified based on their morphology), which show high mitotic activity after settling in the genital ridge and differentiation into oogonia [26, 27]. The decline in proliferation at stage III was, however, accompanied by an increase of the total volume of non-stroma and further small percentage increases in stages IV and V. These were presumably due to increases in the volumes of germ cells from oogonia to oocytes and at the very latter stages activation of follicles.

The highest number of ovarian germ cells is reached around day 90 in the bovine [28] and 182 days in human gestation [29]. The oogonia start to enter meiosis between weeks 10–11 in human [29] and arrest in prophase I until puberty. However in the human fetal ovary, mitotic oogonia occur simultaneously to meiotic oocytes until five months of gestation [29, 30], which is the time point after stage II in our study when the proliferation drops drastically. This is the time when the first primordial follicles are formed, between days 90–140 in the bovine [31] and days 126–133 in the human [32]. However, the numerical density of proliferating cells did slightly increase from stage III to stage IV but not significantly. This is consistent with the activation of primordial follicles toward differentiation into primary follicles, which occurs in the human between 238–266 days [33] and in the bovine between 140–210 days of gestation [31], corresponding to stage III and IV in our study. The subsequent differentiation of the primary follicles to secondary follicles is accompanied by granulosa cell proliferation to form the multiple layers of granulosa cells in more mature follicles. The first secondary follicles have been observed in the bovine fetal ovary after day 210 of gestation [31], which corresponds with stage IV in our study. The numerical density of all the non-stromal cells appeared relatively stable throughout ovarian development (Fig 2E).

### Germ cell markers

**Fig 3:**
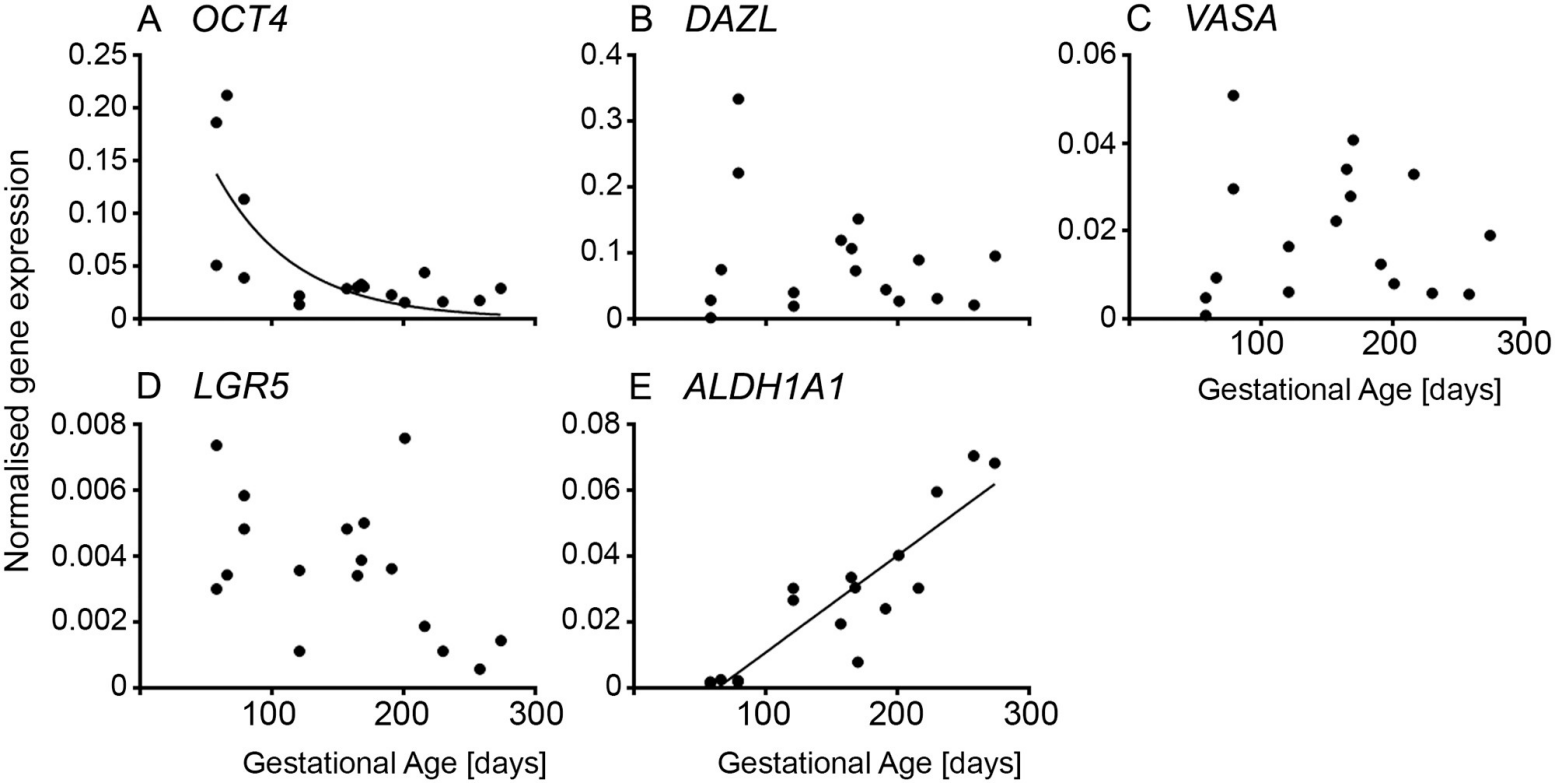
Germ and stem cell-specific genes in fetal ovarian development. Expression levels of genes for each animal are plotted against gestational age in days (n = 16 or 17).

The pluripotency marker *OCT4* was highly expressed in early ovarian development and then declined rapidly by 120 days of gestation at stage II (Fig 2A and S1A Fig). This is expected as germ cells first undergo mitosis and then differentiate and enter meiosis I after colonising the developing ovary. *DAZL* and *VASA* levels were very low at 58 and 66 days, with higher but variable levels during the rest of gestation (Fig 3B and 3D and S1B–S1D Fig). As we observed previously in early gestation in particular [1] the developmental stage of ovaries can differ from one animal to another at the same age. This variability might be caused by environmental effects such as nutrition during gestation [34]. We therefore devised a classification system of developmental stages I to V for the morphometric analysis instead of using gestational age. However, for the RNA analyses the ovaries were not collected for histological classification and so we have analysed those data by age of the fetus as determined by the crown-rump length [24], which could contribute to some variability.

In species with long gestations and large ovaries, like the bovine, different portions of the ovary can be at different developmental stages, especially since follicle formation commences at the medullary-cortical interface and progresses over time in the direction of the surface. Thus it is not unusual to have follicles near the medulla that have not only formed but have commenced growing whilst at the same time ovigerous cords are still present near the surface of the ovary (Fig 3K of [1]). Consequently, although individual germ cells sequentially express *OCT4*, *DAZL* and then *VASA* [1], this does not necessarily occur with RNA analyses of whole ovaries as the isolated RNA from whole ovaries reflects the total of all the developmental stages of germ cells at any one time in each ovary.

### Stem cell markers

Whilst *LGR5* and *ALDH1A1* are stem cell markers, their expression could indicate development of either germ cells or somatic stem cells that exist in the ovary [15]. The stem cell marker *LGR5* declined throughout gestation to low levels in the third trimester (Fig 2B–2D and S1B–S1D Fig). A recent study in human fetal ovaries reported an initial decline in *LGR5* expression levels from 8–11 weeks to 14–16 weeks, but then no further change towards 17–21 weeks [35]. Furthermore, treatment with BMP-4 of cultured fetal cells (~ 14 weeks) increased the expression of *LGR5*. In the mouse, *lgr5* expression first occurs at E13.5 in cells on the surface and subsurface. The subsurface expression declines towards postnatal day 1 and is diminished by postnatal day 7. The *lgr5*-positive cells in the subsurface express *foxl2*, a granulosa cell marker, and the *lgr5*-positive cells on the surface express cytokeratin 8, a surface epithelial marker [36]. *lgr5*-positive cells are also involved in the rapid remodelling of the surface epithelial layer after ovulation, suggesting that they have stem cell properties [36]. On the other hand, Rastetter *et al*. [17] found that *lgr5* is expressed in somatic cells, which neither express *foxl2* nor *nr2f2* (*coup-tfII*), and that *lgr5* expression increases after sex determination in the fetal mouse ovary until birth.

The expression of *ALDH1A1*, another stem cell marker, is barely detectable prior to day 79 but increased significantly and linearly thereafter (Fig 2E and S1E Fig). *ALDH1A1*, encodes retinaldehyde dehydrogenase, and is involved in the last step of the synthesis of retinoic acid, which is crucial for entry of germ cells into meiosis. This would explain the observed increase in the bovine as the entry of oogonia into meiosis occurs around 80 to 130 days of gestation [37]. In contrast, a study in the human fetal ovary analysing the mRNA levels of *ALDH1A1* at 8–9 weeks (undifferentiated primordial germ cells), 14–16 weeks (meiotic entry) and 17–20 weeks (primordial follicle formation), reported a trend of declining expression [12]. The significance of *ALDH1A1* expression in the bovine ovary is yet to be determined.

### GREL cell markers

**Fig 4:**
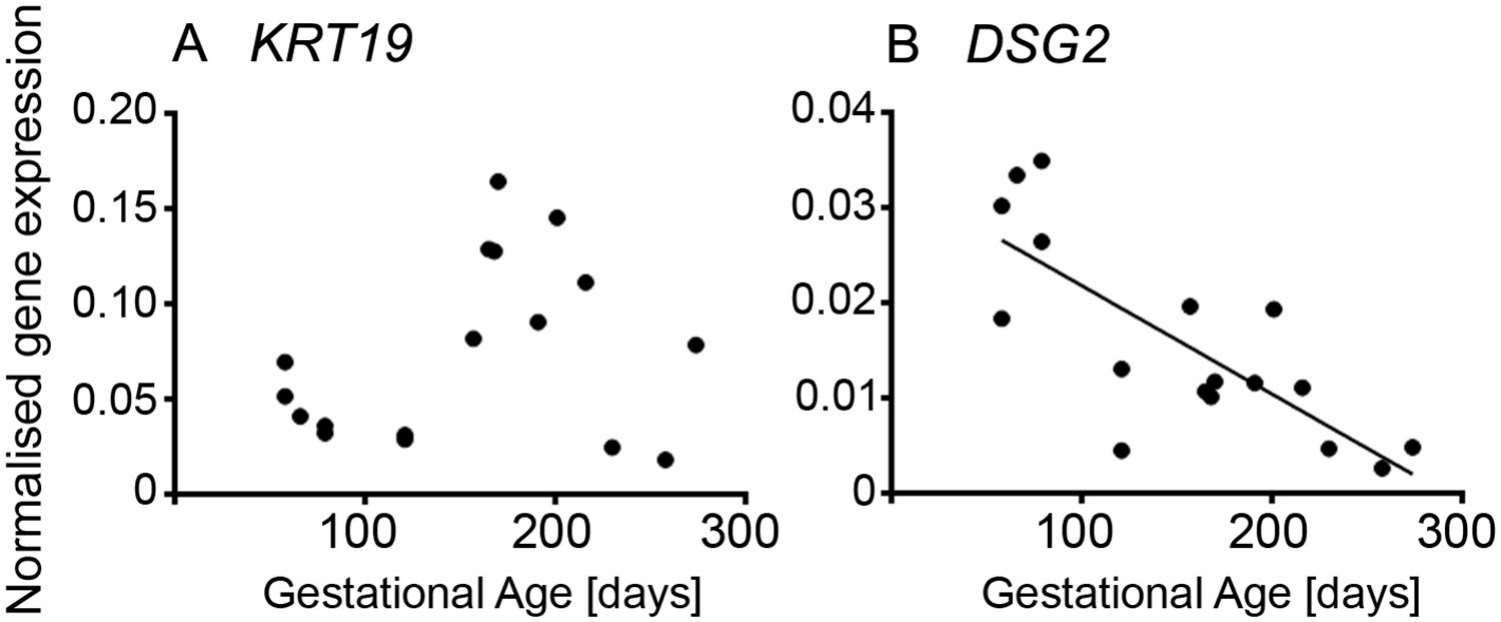
Genes specific for GREL cells in fetal ovarian development. Expression levels of genes for each animal are plotted against gestational age in days (n = 16 or 17).

*KRT19* has been shown to be expressed in GREL cells [1]. Later in development when the surface epithelium has formed it is more highly expressed in the ovarian surface epithelial cells [1]. *KRT19* expression was highest at mid gestation (Fig 4A and S2A Fig). Cytokeratin 19 has been previously detected in the undifferentiated gonadal blastemal, in the somatic cells of ovigerous cords and early follicle stages in the mice [38] and in the rat [39] as well as in the ovigerous cords and primordial, primary and preovulatory follicles in human [40]. *DSG2* is a desmosomal protein also expressed in GREL cells and later in the ovarian surface epithelial cells [1]. It was more highly expressed in early gestation and then declined steadily across gestation (Fig 4B and S2B Fig). This expression pattern can be explained by the differentiation of GREL cells into either surface epithelial cells or granulosa cells with the latter not expressing desmosomes [41].

### Granulosa cell markers

**Fig 5:**
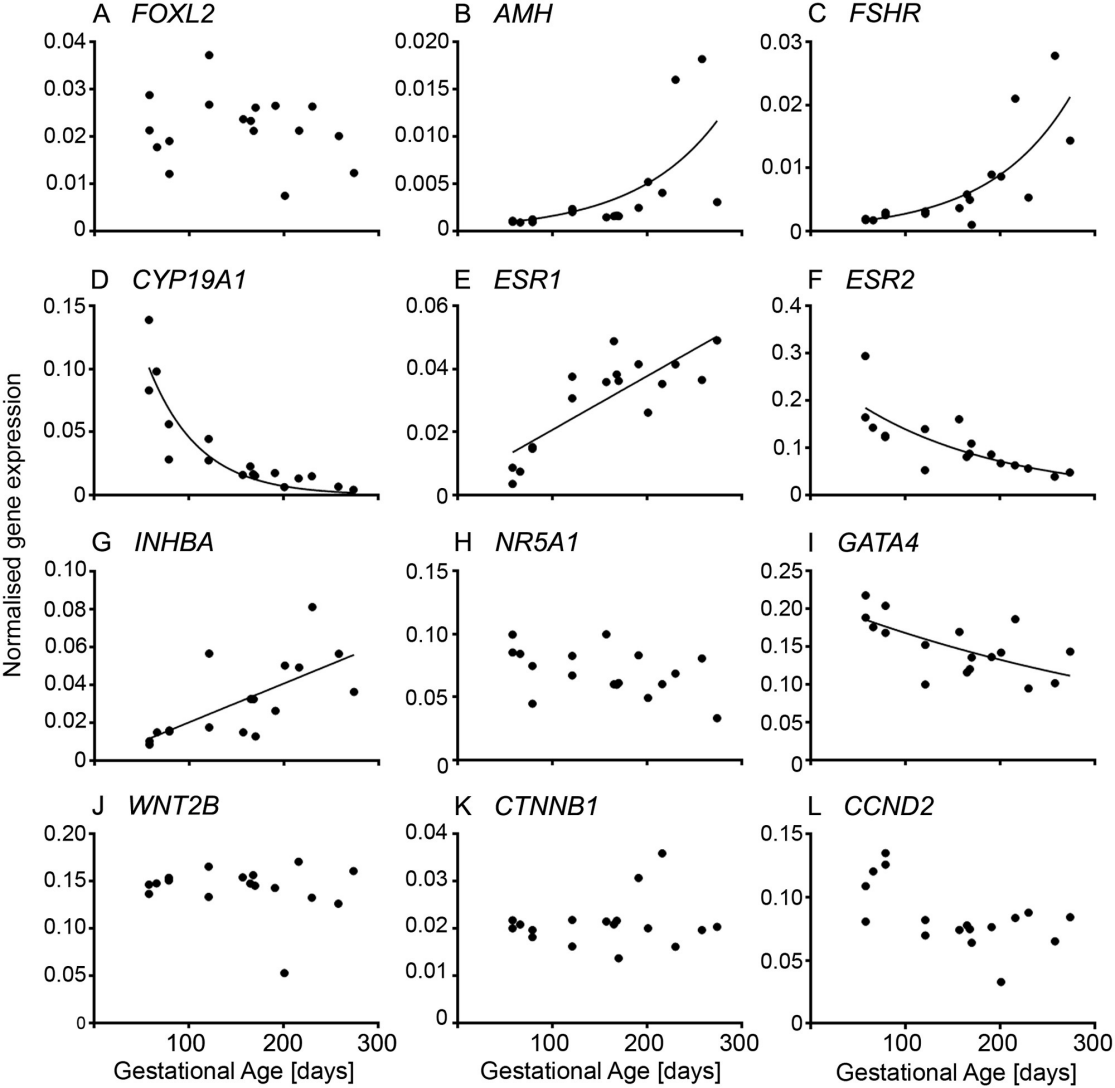
Granulosa cell-specific genes in fetal ovarian development. Expression levels of genes for each animal are plotted against gestational age in days (n = 16 or 17).

The genes analysed in this section were chosen based on knowledge of either direct expression in fetal ovaries or in some cases inferred from expression observed in adult ovarian granulosa cells, such as *CYP19A1*. Analysing the expression of genes specific for granulosa cells revealed three patterns (Fig 4 and S3 Fig). Firstly, genes which increased throughout gestation and these include: *AMH* (Fig 5B and S3B Fig), *FSHR* (Fig 5C and S3C Fig), *ESR1* (Fig 5E and S3E Fig) and *INHBA* (Fig 5G and S3G Fig). *AMH* and *FSHR* have low expression in early and mid-gestation and then a rapid increase towards the end of gestation, whereas the expression of *ESR1* and *INHBA* increased steadily across gestation. The increase in *AMH* as well as *FSHR* is consistent with follicle activation and growth, which commences around day 180 of gestation in the bovine [42]. *AMH* is expressed by granulosa cells from preantral and small antral follicles [43] in women. *FSHR* is expressed in the granulosa cells of primary and secondary follicles with greater expression at antral stages in the bovine fetus [42]. Similar to our findings in this study, *INHBA* increases in second trimester human ovaries [44]. In rodents, *inhba* is expressed at low levels in the fetal ovary [45, 46], which might be due to follicle formation occurring mostly after birth. There is no detectable *INHBA* expression in ovine fetal ovaries throughout gestation [47]. However, the activin/inhibin βA subunit has been immunolocalised to clusters of maturing oocytes in the fetal human ovary (18 weeks) prior to primordial follicle formation, suggesting a role of activin A in the proliferation and survival of germ cells at this stage [44]. Activin-βA mRNA is expressed in the goat from 36 dpc until adulthood and has been considered to be a candidate co-factor for the action of FOXL2 on *CYP19A1* [48]. Treatment of fetal human ovary transplants with activin A increased proliferation of oogonia [44], and in neonatal mice the number of primordial follicles [49]. The corresponding receptors; ActRIIA, ActRIIB and ALK4 (ActRIB) are expressed by germ cells and the latter two additionally in stromal cells or other somatic cells amongst germ cell nests. The signalling targets of activin, Smad 2 and 3, have been localised to the nuclei of stromal cells and other somatic cells between germ cells nests and also to pre-granulosa cells of primordial follicles in human fetal ovaries between 14 and 20 weeks [50]. The expression of *INHBA* is increased in fetal human ovary transplants treated with prostaglandin E2, which in vivo is synthesised by germ cells [51]. Activins not only affect germ cell proliferation and survival, but are also involved in the later stages of follicle differentiation, granulosa cell proliferation and oocyte maturation [reviewed in [52, 53]].

Genes whose expression declined with gestational age included: *CYP19A1* (Fig 5D and S3D Fig), *ESR2* (Fig 5F and S3F Fig), *GATA4* (Fig 5I and S3I Fig) and the cell cycle gene *CCND2* (Fig 5L and S3L Fig). Previous studies in cattle focusing on the expression of P450arom (encoded by *CYP19A1*) and the two oestrogen receptors, ERα (*ESR1*) and ERβ (*ESR2*), throughout gestation, found no decline in the expression of P450arom and ERβ. The results were determined by in situ hybridisation and immunolocalisation [54, 55], neither of which is as accurate as the quantitative RT-PCR used in the current study. Both mRNAs and proteins were expressed by surface epithelial cells, pre-granulosa cells, granulosa cells and cells in the medulla, which are likely stromal cells [54, 55]. On the other hand, it has been reported that oestradiol is produced early in ovarian development and declines when follicle growth occurs later in gestation [31, 56], which could explain the high expression levels we observed in the first trimester and the following observed decline in *CYP19A1* and *ESR2* throughout gestation. In the goat, *CYP19A1* and *ESR2* expression has been detected from 36 dpc and *ESR1* from 40 dpc until adulthood [48, 57]. P450arom is localised to somatic cells, which express *FOXL2*, in the medullar region, whereas ERβ is also localised to germ cells [48]. In mice, *ESR1* is mainly expressed in interstitial (stromal) cells, whereas *ESR2* is expressed in granulosa cells [58]. So, the observed increase of *ESR1* could be due to the expansion of the stromal compartment [13] throughout ovarian development. We previously found that a high cell proliferation rate occurs in stromal and non-stromal compartments early in ovarian development which then declines towards mid- and late gestation [13], which would also explain the higher expression levels of *CCND2* observed early in gestation.

GATA-4 plays an important role during formation of the genital ridge and sex determination [59, 60]. Down-stream targets of GATA-4 are *AMH* [60, 61], *SF1* (alias *NR5A1*), *STAR* (steroidogenic acute regulatory protein), *CYP19A1* [62, 63], *INHA* and *INHBB* [64] and *FOXL2* [65, 66]. It is constantly expressed in the bipotential gonad and after sex differentiation in the ovary in fetal mice [67] and pigs [68]. In the postnatal mice, GATA-4 is found in the granulosa and thecal cells of growing follicles and stromal cells, but not in luteal cells [67], in contrast to the human adult ovary [69]. In human fetal ovaries, *GATA4* is expressed in stromal and pre-granulosa cells and later in granulosa cells with the highest expression occurring early in development (~ 14 weeks) [70]–similar to our findings. Downregulation or loss of GATA-4 caused interruptions in folliculogenesis and recruitment of granulosa and thecal cells resulting in lower numbers of primary follicles and activated follicles, and increased follicular atresia in the existing follicles in adult mouse ovaries [59, 71]. GATA-4 appears to have an anti-apoptotic effect on granulosa cells [70]. A more recent study in the developing mouse ovary suggests that cells expressing *gata4* are precursor cells of granulosa cells and that there are two populations; one which additionally expresses p27, and one which is not expressing p27 and which is therefore highly proliferative [72]. The latter gives rise to the granulosa cells, which will form the so-called cortical follicles from E18.5 to postnatal day 5, whereas the other population is involved in the formation of the medullar follicles between E12.5 and E14.5 [17, 72].

*FOXL2* (Fig 5A and S3A Fig), *NR5A1* (Fig 5H and S3H Fig), *WNT2B* (Fig 5J and S3J Fig) and *CTNNB1* (Fig 5K and S3K Fig) are constitutively expressed throughout gestation. FOXL2 has been described as a female-specific marker from sex differentiation until adulthood [73, 74] and has been shown to be expressed early in bovine ovarian development in the GREL cells which are enclosed together with the germ cells in the ovigerous cords, and in the granulosa cells later in development after follicle formation and growth occur [1]. In the human fetal ovary, *FOXL2* expression increases from 9 weeks to 18 weeks, when the first follicles are formed [32]. The protein localises to the somatic cells streams in the ovarian stroma and the somatic cells intermingled with germ cells, which will become pre-granulosa cells, as well as pre-granulosa cells of primordial follicles. In goats, FOXL2 directly activates *CYP19A1* by binding to its promotor 2 [48] but in the current study no correlation between *FOXL2* and *CYP19A1* was observed, suggesting that there could be differences between species in the regulation of *CYP19A1*. *NR5A1* has been shown to be expressed in the fetal bovine ovary in the cells of the ovigerous cords and on the ovarian surface and later in development in the granulosa cells of growing follicles [1]. In the adult bovine ovary, *NR5A1* (alias *SF-1*) is expressed by granulosa and thecal cells [75]. In contrast to the bovine fetal ovary, *NR5A1* is expressed in the genital ridge of mice and rats until sex determination and then declines in the female until late in gestation in the mice (E18.5) [76] or until after birth in the rat [77] when primordial follicle formation occurs. In the human fetal ovary, persistent *NR5A1* expression has been shown from the point of genital ridge formation up to week 15 of gestation [78] while expression later than this is unclear. Little is known about the expression of *WNT2B* in the ovary. In the immature rat, *WNT2B* has been localised to the ovarian surface epithelium by in situ hybridisation and has also been shown to be expressed in ovarian cancer cell lines [79]. Previously, Hatzirodos et al. [80] found in the bovine adult ovary that *WNT2B* is downregulated in the theca interna of large (9–12 mm) compared to small (3–5 mm) healthy follicles, whereas the Wnt inhibitor *FRZB* was upregulated.

### Relationships between cell markers

Pearson correlation analyses (Tables 2 and 3) of genes expressed in germ and stem cells show a very strong positive correlation between *DAZL* and *VASA*, which both are reported to induce meiotic progression in germ cells [81]. *OCT4* and *LGR5* show both a negative correlation with *ALDH1A1* but a strong positive correlation with the GREL cell marker *DSG2*. *ALDH1A1* was negatively correlated with *DSG2*. There were also correlations between germ cell markers and granulosa cell markers. *OCT4* positively correlated with *CYP19A1* and *CCDN2*, and negatively with *ESR1* and *INHBA*. *DAZL* showed a positive correlation with *CCDN2* and *VASA* but was negatively correlated with *NR5A1*. The stem cell marker *LGR5* correlated negatively with *AMH*, *FSHR* and *INHBA* but positively with *DSG2*. The other stem cell-specific marker, *ALDH1A1*, appears to be strongly correlated with many granulosa cell-specific genes; positively with *AMH*, *FSHR*, *ESR1* and *INHBA*, and negatively with *CYP19A1*, *ESR2*, *GATA4* and *CCDN2*. This is interesting as retinoic acid signalling appears to be essential for induction of meiosis of germ cells but not for granulosa cell specification, at least in mice [82]. On the other hand, *DSG2* was positively correlated with *CYP19A1*, *ESR2*, *GATA4* and *CCDN2*, and negatively with *AMH*, *FSHR*, *ESR1* and *INHBA* and additionally negatively with *ALDH1A1*.

**Table 2:** Pearson’s correlation coefficients between markers for germ and stem cells and all genes examined and gestational age. The intensity of the background colour indicates the strength of the significance of the correlation. Blue is a negative correlation and red is a positive correlation. (^a^*P* < 0.05, ^b^*P* < 0.01, ^c^*P* < 0.01; n = 17).

		Germ and stem cell markers				
		*OCT4*	*DAZL*	*VASA*	*LGR5*	*ALDH1A1*
Gestational age		**-0.620**^**b**^	-0.242	-0.022	-0.479	**0.897**^**d**^
Germ and stem cell markers	***OCT4***		0.055	-0.162	0.375	**-0.560**^**a**^
	***DAZL***	0.055		**0.858**^**c**^	0.284	-0.388
	***VASA***	-0.162	**0.858**^**c**^		0.191	-0.267
	***LGR5***	0.375	0.284	0.191		**-0.603**^**a**^
	***ALDH1A1***	**-0.560**^**a**^	-0.388	-0.267	**-0.603**^**a**^	
GREL cell markers	***KRT19***	-0.197	0.003	0.368	0.394	-0.042
	***DSG2***	**0.779**^**c**^	0.429	0.131	**0.714**^**b**^	**-0.789**^**c**^
Granulosa cell markers	***FOXL2***	-0.160	-0.368	-0.196	-0.297	-0.137
	***AMH***	-0.319	-0.332	-0.367	**-0.506**^**a**^	**0.737**^**c**^
	***FSHR***	-0.314	-0.215	-0.093	**-0.499**^**a**^	**0.702**^**b**^
	***CYP19A1***	**0.651**^**b**^	-0.155	-0.360	0.188	**-0.645**^**b**^
	***ESR1***	**-0.695**^**b**^	-0.14	0.171	-0.476	**0.729**^**c**^
	***ESR2***	0.378	0.008	-0.137	0.351	**-0.712**^**b**^
	***INHBA***	**-0.486**^**a**^	-0.375	-0.292	**-0.573**^**a**^	**0.795**^**c**^
	***NR5A1***	0.315	-0.381	**-0.487**^**a**^	0.053	-0.380
	***GATA4***	0.474	0.346	0.178	0.432	**-0.701**^**c**^
	***WNT2B***	0.169	0.319	0.415	-0.322	-0.212
	***CTNNB1***	-0.039	-0.138	0.011	-0.204	0.011
	***CCND2***	**0.633**^**b**^	**0.597**^**a**^	0.301	0.098	**-0.512**^**a**^

**Table 3:** Pearson’s correlation coefficients between markers for GREL and granulosa cells and all genes examined and gestational age. The intensity of the background colour indicates the strength of the significance of the correlation. Blue is a negative correlation and red is a positive correlation. (^a^*P* < 0.05, ^b^*P* < 0.01, ^c^*P* < 0.01; n = 17).

		GREL cell marker		Granulosa cell markers											
		*KRT19*	*DSG2*	*FOXL2*	*AMH*	*FSHR*	*CYP19A1*	*ESR1*	*ESR2*	*INHBA*	*NR5A1*	*GATA4*	*WNT2B*	*CTNNB1*	*CCND2*
Gestational age		0.276	**-0.775**^**c**^	-0.180	**0.618**^**b**^	**0.733**^**c**^	**-0.794**^**c**^	**0.822**^**c**^	**-0.744**^**c**^	**0.687**^**b**^	-0.441	**-0.622**^**b**^	-0.156	0.175	**-0.587**^**a**^
Germ and stem cell markers	***DAZL***	0.003	0.429	-0.368	-0.332	-0.215	-0.155	-0.140	0.008	-0.375	-0.381	0.346	0.319	-0.138	**0.597**^**a**^
	***VASA***	0.368	0.131	-0.196	-0.367	-0.093	-0.360	0.171	-0.137	-0.292	**-0.487**^**a**^	0.178	0.415	0.011	0.301
	***OCT4***	-0.197	**0.779**^**c**^	-0.16	-0.319	-0.314	**0.651**^**b**^	**-0.695**^**b**^	0.378	**-0.486**^**a**^	0.315	0.474	0.169	-0.039	**0.633**^**b**^
	***LGR5***	0.394	**0.714**^**b**^	-0.297	**-0.506**^**a**^	**-0.499**^**a**^	0.188	-0.476	0.351	**-0.573**^**a**^	0.053	0.432	-0.322	-0.204	0.098
	***ALDH1A1***	-0.042	**-0.789**^**c**^	-0.137	**0.737**^**c**^	**0.702**^**b**^	**-0.645**^**b**^	**0.729**^**c**^	**-0.712**^**b**^	**0.795**^**c**^	-0.38	**-0.701**^**b**^	-0.212	0.011	**-0.512**^**a**^
GREL cell markers	***KRT19***		-0.123	-0.208	-0.323	-0.018	-0.34	0.333	-0.146	-0.107	-0.343	-0.076	-0.204	0.171	**-0.525**^**a**^
	***DSG2***	-0.123		-0.277	**-0.517**^**a**^	**-0.507**^**a**^	**0.587**^**a**^	**-0.820**^**c**^	**0.549**^**a**^	**-0.673**^**b**^	0.27	**0.705**^**b**^	0.011	-0.100	**0.647**^**b**^
Granulosa cell markers	***FOXL2***	-0.208	-0.277		-0.019	-0.248	0.24	0.11	0.306	-0.101	**0.598**^**a**^	-0.137	0.418	-0.062	-0.092
	***AMH***	-0.323	**-0.517**^**a**^	-0.019		**0.624**^**b**^	-0.368	0.317	-0.481	**0.766**^**c**^	-0.011	**-0.563**^**a**^	-0.305	-0.130	-0.277
	***FSHR***	-0.018	**-0.507**^**a**^	-0.248	**0.624**^**b**^		-0.472	0.408	**-0.548**^**a**^	**0.520**^**a**^	-0.211	-0.259	-0.057	0.447	-0.314
	***CYP19A1***	-0.34	**0.587**^**a**^	0.24	-0.368	-0.472		**-0.832**^**c**^	**0.853**^**c**^	**-0.549**^**a**^	**0.581**^**a**^	**0.632**^**b**^	0.111	-0.085	0.439
	***ESR1***	0.333	**-0.820**^**c**^	0.11	0.317	0.408	**-0.832**^**c**^		-0.733	**0.550**^**a**^	-0.410	**-0.754**^**c**^	0.089	0.121	**-0.531**^**a**^
	***ESR2***	-0.146	**0.549**^**a**^	0.306	-0.481	**-0.548**^**a**^	**0.853**^**c**^	**-0.733**^**c**^		**-0.721**^**b**^	**0.626**^**b**^	**0.748**^**c**^	0.152	-0.122	0.297
	***INHBA***	-0.107	**-0.673**^**b**^	-0.101	**0.766**^**c**^	**0.520**^**a**^	**-0.549**^**a**^	**0.550**^**b**^	**-0.721**^**b**^		-0.339	**-0.699**^**b**^	-0.360	0.120	-0.424
	***NR5A1***	-0.343	0.270	**0.598**^**a**^	-0.011	-0.211	**0.581**^**a**^	-0.410	**0.626**^**b**^	-0.339		0.240	0.118	0.036	0.068
	***GATA4***	-0.076	**0.705**^**b**^	-0.137	**-0.563**^**a**^	-0.259	**0.632**^**b**^	**-0.754**^**c**^	**0.748**^**c**^	**-0.699**^**b**^	0.240		0.238	0.223	**0.512**^**a**^
	***WNT2B***	-0.204	0.011	0.418	-0.305	-0.057	0.111	0.089	0.152	-0.360	0.118	0.238		0.185	**0.555**^**a**^
	***CTNNB1***	0.171	-0.100	-0.062	-0.130	0.447	-0.085	0.121	-0.122	0.120	0.036	0.223	0.185		-0.052
	***CCND2***	**-0.525**^**a**^	**0.647**^**b**^	-0.092	-0.277	-0.314	0.439	**-0.531**^**a**^	0.297	-0.424	0.068	**0.512**^**a**^	**0.555**^**a**^	-0.052	

Surprisingly, no correlation existed between *KRT19* and *DSG2*. Furthermore, *KRT19* was only correlated to *CCDN2*. Correlation analysis of the genes within the group of granulosa cell-specific markers, revealed a positive correlation between *FOXL2* and *NR5A1*, between *AMH*, *FSHR* and *INHBA*, between *FSHR* and *ESR2*, and between *WNT2B* and *CCDN2*. *CYP19A1* correlated positively with *ESR2*, *NR5A1* and *GATA4*, but negatively with *ESR1* and *INHBA*. As expected, *ESR1* was positively correlated with *INHBA*, but negatively with *ESR2*, *GATA4* and *CCDN2*. *ESR2* showed a positive correlation with *GATA4*, whereas *INHBA* was negatively correlated with *GATA4*. Additionally, *GATA4* was positively correlated with *CCDN2*. Interestingly, *CTNNB1* showed no correlation with any of the germ, stem, GREL or granulosa cell markers.

The multitude of both negative and positive correlations, therefore, in expression of genes in germ and somatic cells, and the correlations amongst genes expressed within a cell lineage, suggest a degree of co-regulation or coordination of behaviours of different cells. Since many of these genes either increase or decrease during gestation, the relationships between these genes may reflect their physiological role in the transition of ovigerous cords to follicles, oogonia to oocytes or GREL cells to granulosa cells.

#### Gene expression and ovarian development

The early cortex is characterised by formation and growth of the ovigerous cords which occurs by replication of GREL and germ cells. It is characterised by high proliferation indices (Fig 2) and high expression of *CCND2* (Fig 5). The germ cells are less mature and there is high expression of *OCT4* and *DAZL*. GREL cells are present at that stage and they have extensive cell junctions and express *DSG2* which is elevated at these early stages (Fig 4). *CYP19* and *ESR2* are also highly expressed at these early stages suggesting that oestradiol might have a role then. Subsequently the cords begin to break into smaller clusters of cells (stage II) and *ESR1* and *ALDH1* are upregulated. The change in *ESR1* probably reflects maturation of oogonia into oocytes. As development continues and follicles are being formed (stage III), cell replication declines and *CYP19A1*, *ESR2* and *OCT4* are down regulated. This, in conjunction with continued expansion of stroma [13], may have led to a decline in the proportion of the non-stromal compartments in the cortex. The changes in *OCT4* signal maturation of the germ cells. At later stages follicle activation commences and expression of genes in growing follicles, *FSHR*, *AMH* and *INHBA*, is increased. *KRT19* is expressed in the surface epithelium [1] and its expression is increased as the surface epithelium is formed (stage IV).

## Conclusions

The expression of many genes increased across gestation matched by a concomitant decline in others, with many being positively or negatively correlated, even when potentially not expressed in the same cell type. These relationships between genes may reflect their roles in the transition of ovigerous cords to follicles, oogonia to oocytes, or GREL cells to granulosa cells. Further work is now underway to identify genes that act as regulators and those that are regulated.

## Supporting information

S1 Fig Expression of germ and stem cell markers. Measurement of gene expression of germ and stem cell markers in fetal ovaries by q-PCR graphed by trimester (1st, 2nd and 3rd trimesters have n = 5, n = 7 and n = 5 animals, respectively). Mean ± SEM are shown and statistical differences between trimesters are shown as **, or ****, indicating *P* < 0.01 or *P* < 0.0001, respectively.
(TIF)

S2 Fig Expression GREL cell markers. Measurement of gene expression of GREL cell markers in fetal ovaries by q-PCR graphed by trimester (1st, 2nd and 3rd trimesters have n = 5, n = 7 and n = 5 animals, respectively). Mean ± SEM are shown and statistical differences between trimesters are shown as ***, indicating *P* < 0.001, respectively.
(TIF)

S3 Fig Expression of granulosa cell markers. Measurement of gene expression of granulosa cell markers in fetal ovaries by q-PCR graphed by trimester (1st, 2nd and 3rd trimesters have n = 5, n = 7 and n = 5 animals, respectively). Mean ± SEM are shown and statistical differences between trimesters are shown as *, **, ***, or ****, indicating *P* < 0.05, *P* < 0.01, *P* < 0.001 or *P* < 0.0001, respectively.
(TIF)
