# Supplementary figures and images for: Morphometric analyses and gene expression related to germ cells, gonadal ridge epithelial-like cells and granulosa cells during development of the bovine fetal ovary
Source: PLoS One. 2019 Mar 22;14(3):e0214130. doi: 10.1371/journal.pone.0214130 (PMC6430378; doi:10.1371/journal.pone.0214130)

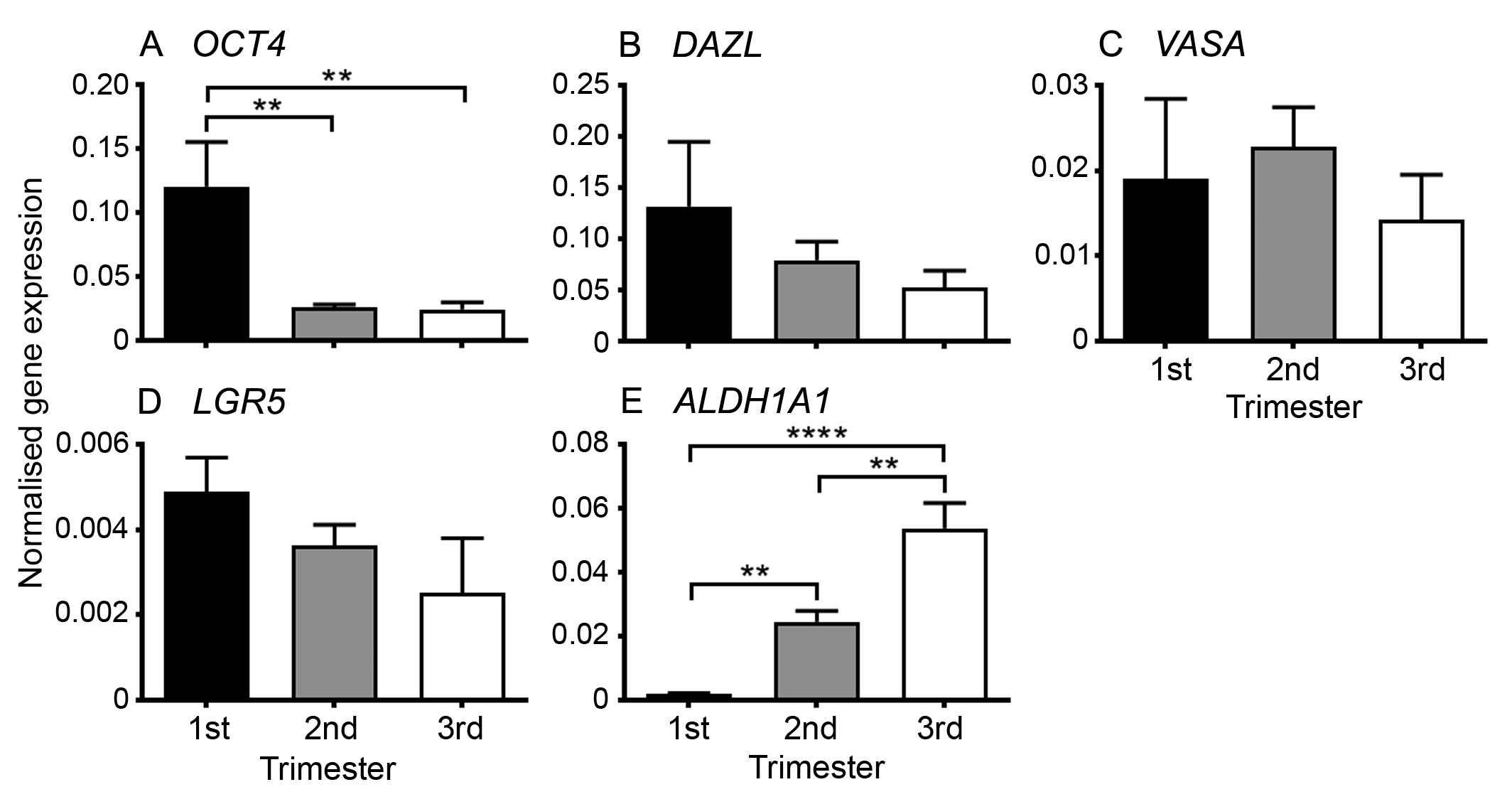

Supplement: S1 Fig — Measurement of gene expression of germ and stem cell markers in fetal ovaries by q-PCR graphed by trimester (1st, 2nd and 3rd trimesters have n = 5, n = 7 and n = 5 animals, respectively). Mean ± SEM are shown and statistical differences between trimesters are shown as **, or ****, indicating P < 0.01 or P < 0.0001, respectively. (TIF) [file pone.0214130.s001.tif]

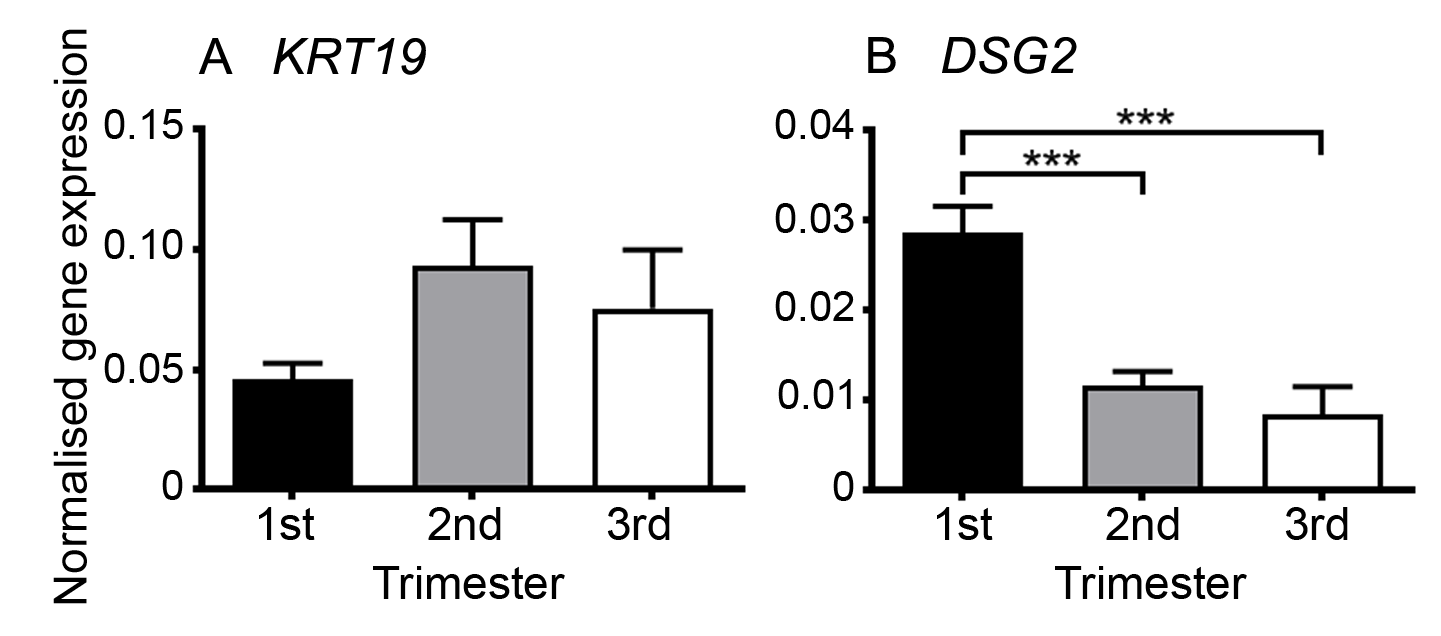

Supplement: S2 Fig — Measurement of gene expression of GREL cell markers in fetal ovaries by q-PCR graphed by trimester (1st, 2nd and 3rd trimesters have n = 5, n = 7 and n = 5 animals, respectively). Mean ± SEM are shown and statistical differences between trimesters are shown as ***, indicating P < 0.001, respectively. (TIF) [file pone.0214130.s002.tif]

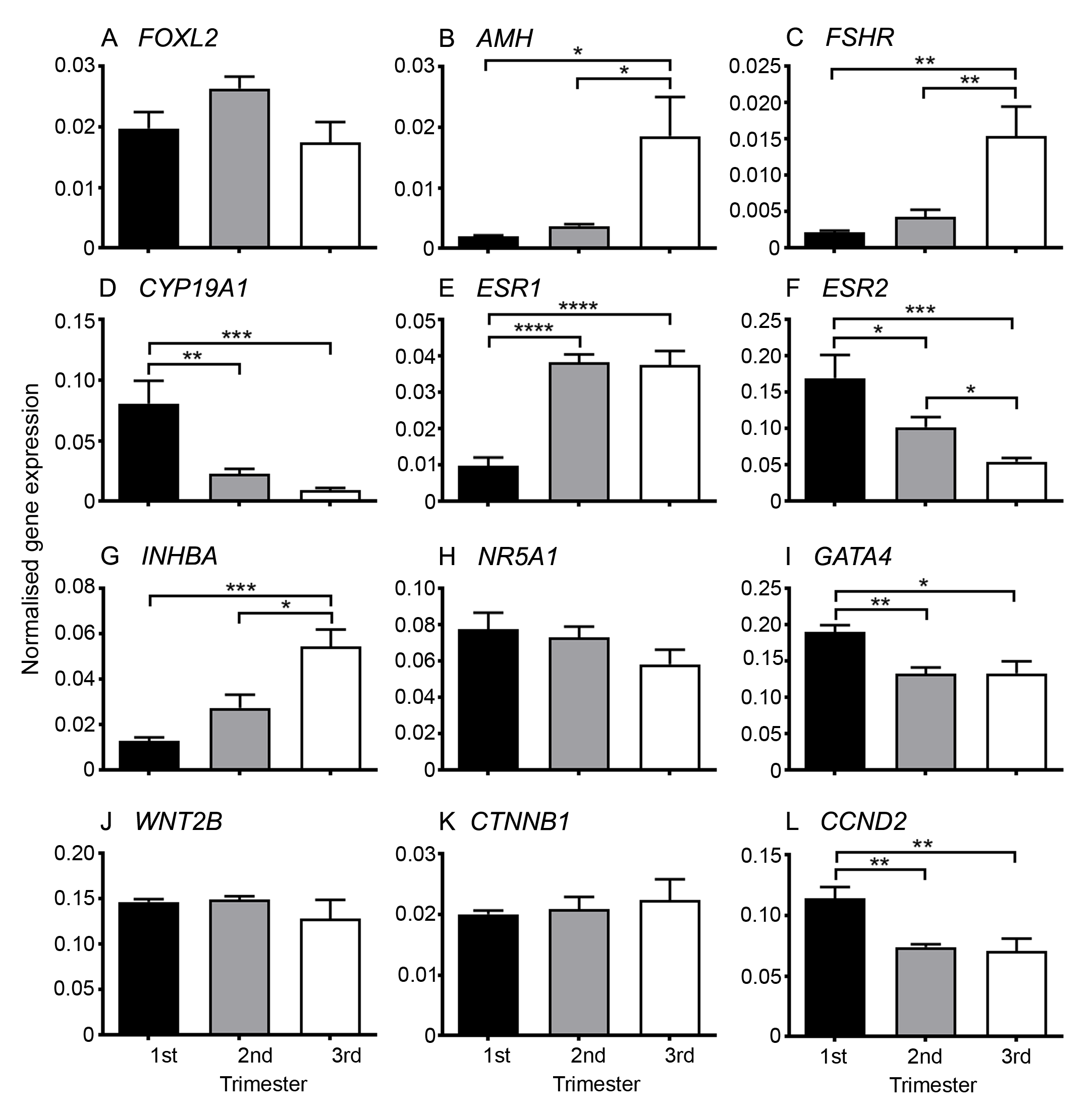

Supplement: S3 Fig — Measurement of gene expression of granulosa cell markers in fetal ovaries by q-PCR graphed by trimester (1st, 2nd and 3rd trimesters have n = 5, n = 7 and n = 5 animals, respectively). Mean ± SEM are shown and statistical differences between trimesters are shown as *, **, ***, or ****, indicating P < 0.05, P < 0.01, P < 0.001 or P < 0.0001, respectively. (TIF) [file pone.0214130.s003.tif]
